# Bioaccumulation of Trace Metals in Mussel (*Mytilus galloprovincialis*) from Mali Ston Bay during DSP Toxicity Episodes

**DOI:** 10.3390/molecules200713031

**Published:** 2015-07-17

**Authors:** Ivana Ujević, Nenad Vuletić, Jelena Lušić, Nikša Nazlić, Grozdan Kušpilić

**Affiliations:** 1 Laboratory of Plankton and Shellfish Toxicity, Institute of Oceanography and Fisheries, Šetalište I. Meštrovića 63, P.O. Box 500, 21000 Split, Croatia; E-Mail: nazlic@izor.hr; 2 Faculty of Sciences, University of Split, Teslina 12, 21000 Split, Croatia; E-Mail: nenov@net.hr; 3 Laboratory of Chemical Oceanography and Sedimentology of the Sea, Institute of Oceanography and Fisheries, Šetalište I. Meštrovića 63, P.O. Box 500, 21000 Split, Croatia; E-Mails: lusic@izor.hr (J.L.); kuspe@izor.hr (G.K.)

**Keywords:** trace metals, shellfish toxicity, bioaccumulation, mussel, DSP, diarrhetic shellfish poisoning

## Abstract

The Croatian National Monitoring Program revealed the presence of Diarrhetic Shellfish Poisoning (DSP) toxicity in Mediterranean blue mussel (*Mytilus galloprovincialis*) from breeding farms in southern Adriatic Sea through January to June 2011. The mouse bioassay tests (MBA; at the time the official method for DSP toxins) were accompanied by atypical symptomatology in the animals and this caused doubts about the assay results. Consequently, in parallel studies reported here, the concentration of Cd, Cr, Cu, Ni, Pb and Zn in soft tissue of DSP positive and negative mussels samples was determined. Cd, Cr, Zn and Ni show higher values in approximately 75% of the DSP positive samples, whereas for Pb and Cr the values were 26% and 34%, respectively. This trend was unchanged during the whole observation period.

## 1. Introduction

The sea is a part of the environment that has a significant role in the economy and at the same time represents a very complex ecosystem that is under a constant influence of various human activities. Marine organisms have an ability to absorb and accumulate various contaminants from the water, and they have been long used as biological indicators of marine pollution or as model organisms for studies of dietary sources of certain contaminants and natural toxins [1,2,3]. Accumulation of trace metals and marine biotoxins in shellfish are studies that have mainly been carried out separately. However, some studies indicate the role of trace metals and emerging toxins, gymnodimines (GYMs) and spirolides (SPXs), on the false positive results of mouse bioassay test (MBA) for marine biotoxins [4,5,6].

Some studies have been conducted during the last decade on trace metal contamination in mussels from the Croatian coastal area in the eastern Adriatic Sea [7,8,9] and according to the obtained data, contamination of mussels is restricted to land-based sources of contamination. Metal levels found in Croatian mussels lie within the range of metal concentrations determined in other low to moderately polluted Adriatic and Mediterranean areas [10,11,12,13,14,15,16]. Strong affinity of metal ions to organic ligands allows them to be transported through the membrane by binding to transport proteins and form a concentration gradient of free metal ions through the cell membrane. Trace metals may also affect the microalgal production of marine biotoxins [6], as the production of biotoxins was enhanced by the addition of particular trace metals to specific microalgae. The effects of trace metals on biotoxin production are of concern given the substantial road and land runoff inputs to coastal waters and subsequent increase in algal bloom toxicity that could greatly impact shellfish aquaculture.

The presence of GYMs and SPXs in Croatian mussels was detected for the first time in 2006 [17], and was also confirmed in shellfish samples from central and southern Adriatic Sea during the DSP toxicity study in 2011 [18]. GYMs and SPXs are emerging lipophilic marine natural toxins that belong to a heterogeneous group of macrocyclic compounds called cyclic imines. Since their discovery in the early 1990s, GYMs and SPXs have been demonstrated to have a global distribution range, including the Adriatic Sea [19]. These toxins are well known due to their “fast acting toxicity” in an MBA test. GYMs and SPXs have not yet been linked to human poisoning outbreaks, however, their true risk has not been properly assessed due to the scarce exposure data and limited toxicology records. In a recent investigation carried out on Caco-2 cells, it was assessed that SPX could be absorbed in the human intestinal epithelium [20]. To date, these toxins have not been included in the official shellfish monitoring, however, their existence can lead to incorrect results when applying an MBA. Besides, a mouse bioassay may give false positive results in the presence of other substances than biotoxins, such as higher contents of zinc or fatty acids [4,5,21,22,23,24].

The Croatian shellfish industry has been affected by the statutory closure of Mali Ston Bay in 2011, following the analysis of samples causing rapid reactions in the regulatory body approved test for Diarrhetic Shellfish Poisoning (DSP) toxins, the MBA test. It is supposed that the atypical results obtained were due to procedural problems of the MBA test or other compounds co-extracted when preparing shellfish samples for this test. The objective of this study was to determine trace metal concentrations (Cd, Cr, Cu, Ni, Pb and Zn) in DSP positive and negative mussel samples collected under the Croatian National Monitoring Program in the January to June 2011 period, in order to define a possible relationship between positive MBA test results and trace metal concentrations.

## 2. Results and Discussion

### 2.1. Correlation between MBA DSP Results and Trace Metal Concentrations in Mussels

The concentrations of trace metals (Cd, Cr, Cu, Ni, Pb and Zn) were analysed in 132 samples (19 DSP negative and 113 DSP positive samples) of blue mussels (*Mytilus galloprovincialis*) collected from farms located in the southern Adriatic Sea on a weekly basis or more frequently if the results of MBA tests were positive (but not less than 48 h).

**Figure 1:**
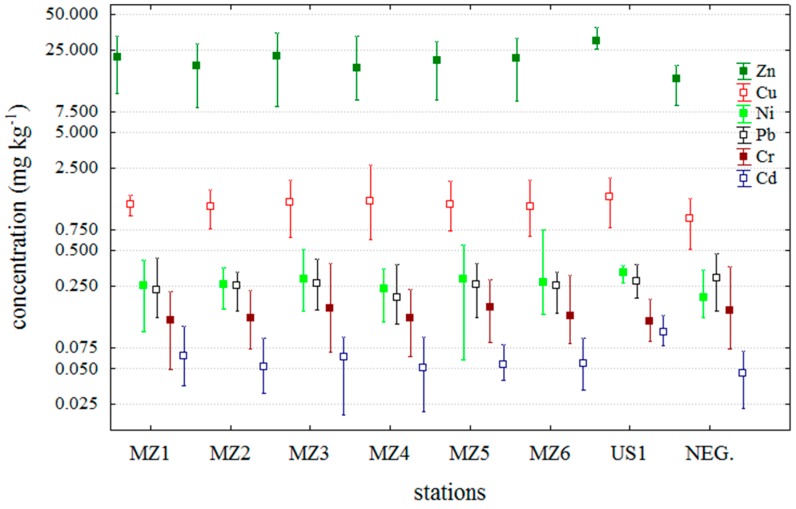
Metal concentration in studied blue mussels. NEG–DSP negative mussels samples; other samples are DSP positive.

Means, minimums and maximums of trace metals that have been analysed in this study are shown in Figure 1. Trace metal results for the negative samples are separated in the graph to emphasize the differences between sample batches. Trace metal concentrations are expressed per wet weight (w wt).

Cadmium concentrations in the DSP positive samples lay within the range of 0.0216 mg·kg^−1^ to 0.1403 mg·kg^−1^. Mean values for each station as well as 72.5% of all DSP positive results were higher than the mean values pertaining to Cd levels in DSP negative samples (Figure 1). The mean Cd concentration for all DSP positive samples was 0.0590 mg·kg^−1^, while for the DSP negative samples it was recorded to be a 22.5% lower (0.0457 mg·kg^−1^) value. Cadmium concentrations lay in a similar range at all stations except for the station US1 that is a location with greater exposure to maritime traffic (Figure 1).

Lead concentrations in DSP positive samples lay within the range of 0.1185 mg·kg^−1^ to 0.4325 mg·kg^−1^. The mean concentration of Pb was 0.2451 mg·kg^−1^ for DSP positive, whereas an 18.7% higher mean, 0.2910 mg·kg^−1^, was recorded in DSP negative mussels. Lead bioaccumulation is opposite to that of Cd in respect to DSP results, as confirmed by the fact that 74% of DSP positive samples have lower concentrations than it is the mean concentration for DSP negative samples. Bioaccumulation of lead, like the other trace metals, is dependent on the total amount of metal forms available for the absorption process by organisms, as well as their uptake and excretion mechanisms. Circulation of Pb in the sea, including lower transport levels in mussels during the DSP toxicity reveals that, unlike Cd, Pb does not affect potential production of marine biotoxins.

Chromium showed a distribution pattern similar to that of Pb for positive and negative DSP samples from the investigated area (Figure 1). The mean Cr concentration for each station, except for MZ5 and MZ3, was lower than the mean values for DSP negative samples. Cr concentrations in the DSP positive mussels lay within the range of 0.0492 mg·kg^−^^1^ to 0.3892 mg·kg^−^^1^. The mean Cr concentration values in DSP positive and negative mussel samples were 0.1441 and 0.1542 mg·kg^−1^, respectively. However, 66.3% of the values recorded in DSP positive samples were higher than the mean value pertaining to Cr levels in DSP negative samples.

Nickel concentrations in DSP positive mussels lay within the range of 0.0588 mg·kg^−^^1^ to 0.7452 mg·kg^−^^1^, and in 77.6% DSP positive samples Ni concentration was higher than the mean Ni concentration of DSP negative samples, that is in consistency with the results obtained for Cd. The mean Ni concentration in DSP positive and negative samples was 0.2691 mg·kg^−1^ and 0.2002 mg·kg^−1^ (it is 25.6% lower), respectively.

Copper concentrations in DSP positive mussels lay within the range of 0.6215 mg·kg^−1^ to 2.6732 mg·kg^−1^. The mean Cu concentration in DSP positive samples was 1.2394 mg·kg^−1^ and 0.9352 mg·kg^−1^ in DSP negative ones. The ratio between the mean concentration values of DSP positive and negative samples is equal to the ratio pertaining to cadmium and nickel concentrations (Figure 1) as well as the percentage of DSP positive samples (75.5%) in which were recorded higher concentrations than was the mean Cu concentration of DSP negative samples.

Zinc concentrations in DSP positive mussels lay within the range of 8.1182 mg·kg^−1^ to 38.9059 mg·kg^−1^. The mean Zn concentration values in DSP positive and negative mussel samples were 20.7525 and 15.6578 mg·kg^−1^ (it is 24.6% lower), respectively. In addition, 73.5% of the values recorded in DSP positive samples were lower than the mean value pertaining Zn levels in DSP negative samples. The distribution of Zn concentrations was equal to those of Cd, Cu or Ni (Figure 1).

All analysed mussel samples were collected through the official Croatian National Monitoring Programme for marine biotoxins, the ASP toxins were analysed by HPLC, while the DSP and PSP toxins were analysed by MBA. According to the food control requirements provided by the EU Directive weekly analyses showed satisfactory results for ASP and PSP, but not for the DSP toxins [25]. The ASP toxins (domoic and epi-domoic acids) lay below the limit of detection (LOD) and all mussel samples responded negatively to PSP MBA tests. Most of the MBA positive samples for DSP were recorded in June 2011 and were characterized by an acute toxicity reaction in animals with atypical symptomatology for DSP toxicity. The latter consisted of neurological signs, convulsions and cramps within a few minutes after injection, and subsequent death within a few to 30 min. However, the animals that survived fully recovered and behaved normally. In order to detect the specific toxins that caused this DSP toxicity, analyses of the lipophilic toxins (OA—okadaic acid, DTXs—dinophysistoxins, AZA-1, AZA-2 and AZA-3—azaspiracids, YTXs—yessotoxins and the unregulated GYMs and SPXs) in methanol shellfish extracts were also performed by LC-MS/MS (the results were presented on the International Conference on Harmful Algae, Wellington, New Zealand, 27–31 October 2014). Since this analysis did not reveal any EU Directive-regulated lipophilic toxins, except for low concentrations of gymnodimine and spirolide (in the range of 5 to 15 μg·kg^−1^) found in most of the DSP positive shellfish samples, there is reasonable doubt that this is the cause of the MBA positive tests [18]. Given that the comparison of trace metal concentrations in DSP positive and negative samples reveals (in contrast with lower Pb and Cr levels) higher Zn, Cd, Cu and Ni levels in >70% DSP positive samples (Figure 1), it could be assumed that the occurrence of biotoxins, (GYMs and SPXs) is correlated to Cd, Ni, Cu and Zn, but not to Pb and Cr.

As phytoplankton communities (producers of marine biotoxins) responsd relatively fast to water column changes impacted by various sources, whether natural or anthropogenic, variations in the amount of trace metals affect metabolic processes in phytoplankton cells. Increases of trace metal content on a small temporal or spatial scale enhances the production of marine biotoxins by phytoplankton cells. Trace metal effects on the production of marine biotoxins was investigated by Rhodes *et al.* [6]. The production was enhanced by the addition of particular trace metals to specific microalgae. An increase in domoic acid isomer-C production by *Pseudo-nitzschia australis* was achieved by adding copper or zinc (0.1–0.2 µmol·L^−1^) to standard growth media. Copper addition also resulted in an increase of 100% in the production of palytoxin-related compounds by *Ostreopsis siamensis* and a 50% increase in okadaic acid diol ester production by *Prorocentrum lima.* Another explanation for the role of trace metals in marine biotoxin accumulation in shellfish includes facilitated transport processes of biotoxins to shellfish tissue that participates in enhanced metal bioaccumulation.

### 2.2. Temporal Distribution of Trace Metals during the Period of DSP Shellfish Toxicity

**Figure 2:**
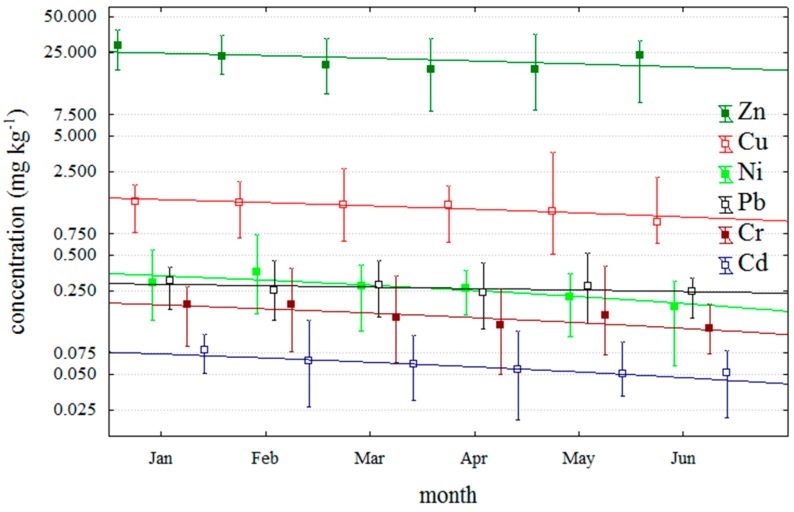
Metal concentration in studied blue mussels on the period from January to June 2011. All samples are DSP positive.

This study examined small-scale trends in investigated trace metals for aquacultured DSP positive mussel over six month in 2011 (Figure 2). Trace metal concentrations of all DSP positive samples exhibited relatively similar decreasing trends from January to June. These results coincide with the results of other authors who studied seasonal changes in metal contents in the whole soft tissue of mussels, genus *Mytilus*, in moderate climates of the Northern Hemisphere [26,27,28,29].

The authors concluded that seasonal variations of trace metal concentrations in shellfish soft tissue take the form of a sinusoid curve with the metal concentrations generally being the highest in the coldest part of the year (late winter and early spring), and the lowest during the summer and early fall [29]. Therefore, biological availability of trace metals is highly dependent on seasonal meteorological factors which affect their concentration and physical and chemical forms in the sea water, as well as the metabolic rate of mussels, especially in shallow coastal areas up to 10 m deep where the influence of waves, wind and nutrients is the most intensive.

## 3. Experimental Section

**Figure 3:**
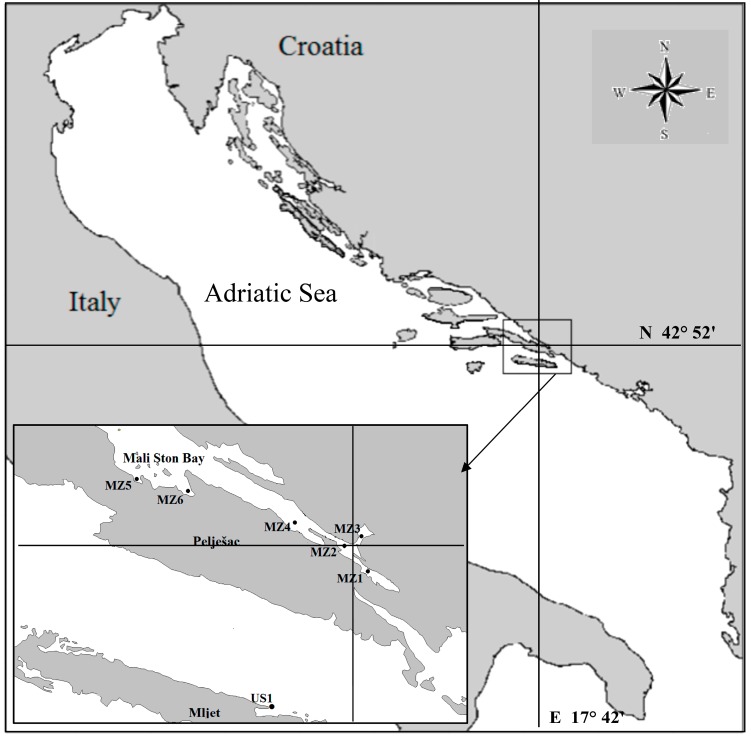
Map of ali Ston Bay and location of sampling stations.

Mussel samples (*Mytilus galloprovincialis*) for the investigation were collected within the scope of the official control analyses at seven control stations, six of which are located in the Mali Ston Bay and one on the island of Mljet (Sobra Bay; US1), from January to June 2011 (Figure 3). Mali Ston Bay, the most important area of shellfish farming in Croatia with a tradition of more than one hundred years in length, is located on the far south of the Croatian Adriatic coast.

Samples were collected weekly or more frequently (but not less than bi-daily) during the first part of the year when a large number of samples showed DSP positive MBA tests. In order to obtain a representative sample, 4 kg of mussels was collected at each sampling. The whole soft tissue was separated from mussel shells and homogenized. The samples for trace metal analysis were pulled from 300–350 g of homogenized frozen soft tissue. Approximately 10 g of each frozen tissue was lyophilized by a Lio 5P freeze-dryer (Martin Christ, Osterode am Harz, Germany) and subsequently ground and homogenized to a fine powder. Microwave assisted acid digestion of samples for trace metal analysis was carried out by a modified US EPA 3052 method [30]. The procedure involved a mixture of acids: HNO_3_ (68%, Trace analysis grade, Fisher Scientific UK, Loughborough, UK,), HClO_4_ (65%, Trace analysis grade, Fisher Scientific UK), and H_2_O_2_ (30%, Merck, Darmstadt, Germany). Approximately 0.25 g of tissue sample was placed in a Teflon vessel and treated with a 2:1 mixture of HNO_3_ and HClO_4_. Samples in closed vessels were subjected to several heating steps in the microwave oven, followed by an acid evaporation step on a hot plate. Finally, samples were treated with a mixture of HNO_3_ and H_2_O_2_ (1:1) and underwent a final microwave oven digestion step followed by sample dilution with deionized water up to 15 mL [30].

Analysis of trace metal concentration in digested samples was performed on an atomic absorption spectrometer (Analyst 800, Perkin-Elmer, Shelton, CT, USA) using the graphite furnace (Cd, Cr, and Pb) or flame (Cu, Ni and Zn) atomic absorption techniques. The instrument was calibrated with aqueous standard solutions prepared from commercially available stock standard solutions of ultrapure grade, supplied by Merck. The precision and accuracy of the applied analytical method were assessed by analysis of a standard reference material of marine biota sample (SRM 2976, freeze-dried mussel tissue, National Institute of Standards and Technology, Gaithersburg, MD, USA). The obtained results were in good agreement with the certified values for all metals and the standard deviations were low, proving good repeatability of the method. Recovered values (mean% recovery ± SD) were: 97.3% ± 7.5% for Cd, 101.6% ± 6.5% for Cr, 105.5% ± 4.6% for Cu, 108.5% ± 3.6% for Ni, 103.5% ± 7.3% for Pb and 105.6% ± 6.0% for Zn.

Deionised water with a Milli-Q Water Purification System (Merck Millipore, Molsheim, France) was used for cleaning glass, plastic and teflonware, preparation of stock solutions and dilution of samples. Glass and plastic ware used for tissue analysis were cleaned with 5% solution of nitric acid for 48 h and rinsed with deionised water.

## 4. Conclusions

Examination of trace metal distribution patterns in DSP positive and negative aquacultured mussels reveals two types of distributions. Those of Cd, Cu, Ni and Zn were characterized with higher concentration in more than 72% of DSP positive samples compared to the mean value of DSP negative mussel samples. Conversely, concentrations of Pb and Cr were 74% and 66.3% lower, respectively, compared to the results of DSP negative mussels. Previous analysis of investigated samples, carried out for lipophilic toxins, revealed only low concentrations of gymnodimine (GYM) and spirolide (SPX) in DSP positive samples. For that reason our findings indicate that production of GYM and SPX may be correlated to Cd, Cu, Ni and Zn, but not to Cr and Pb, and also, this study calls for further research of physiological interactions of biotoxins, especially lipophilic toxins and trace metals.
